# Distance to landfill and human activities affects the debris incorporation into the white stork nests in urbanized landscape in central Spain

**DOI:** 10.1007/s11356-020-09621-3

**Published:** 2020-06-15

**Authors:** Zuzanna Jagiello, Alejandro López-García, José I. Aguirre, Łukasz Dylewski

**Affiliations:** 1 Institute of Zoology, Poznań University of Life Sciences, Wojska Polskiego 71C, 60-625 Poznań, Poland; 2 Department of Biodiversity, Ecology and Evolution, Complutense University of Madrid, José Antonio Novais, 12, 28040 Madrid, Spain; 3 Institute of Dendrology, Polish Academy of Sciences, Parkowa 5, 62-035 Kórnik, Poland

**Keywords:** Birds, Human pressure, Landfill, Nest, Plastic pollution

## Abstract

Human’s activities dominates many aspects of the Earth’s environment; thus animals are forced to adapt and respond to the resulting changes in habitat structure and functioning due to anthropogenic pressure. Along with the growing human population and the associated amount of waste produced, the amount of different type of physical contamination component in environment is increasing. Incorporation of debris in nests may be a mounting avian response to anthropogenic pollution. In this research, we quantified the constituent pieces and total mass of human-derived materials incorporated in white stork nests. The study was conducted on four locations in central Spain where white storks nest along a urbanization gradient. In total, we examined 49 nests. This study demonstrates that the incorporation of debris by white storks into their nests is related to human activity, measured by the Human Footprint Index (HFI). Moreover, the distance between these nests and landfills predicts the occurrence of debris incorporated into nests. Our study shows that birds nest building behaviour is impacted by human activities and pollution in environment.

## Introduction

Currently, 75% of the Earth’s surface had experienced measureable human pressures, i.e. commonly related to activities of humans which cause a negative effect towards biodiversity (Venter et al. 2016; Jones et al. 2018). Human activities lead to habitat fragmentation, conversion of natural habitats to production land uses, introduction of exotic species and have caused biodiversity losses (Cardinale et al. 2012; Hooper et al. 2012), also the spread of invasive alien species (Lowe et al. 2000), and climate change (Walther et al. 2002; Kalnay and Cai 2003) on a global scale and at a rapid rate. Nature in general (and particularly the animal kingdom) is forced to respond and adapt to the resulting changes in environment (Lowry et al. 2012).

In this context, researchers have developed tools to measure the impact of human activities on Earth surface (Jones et al. 2018). One of these tools is the Human Footprint Index (HFI) which is a proxy for human population density, settlements, crop/pasture land, roads, and other access points, night-time lights, and incorporates factors such as the size and remoteness of a given area (Sanderson et al. 2002; Venter et al. 2016; Jones et al. 2018). Human Footprint Index is a global map, a dataset of 1-km indexed grid cells which covers all continents except Antarctica and remote islands (Sanderson et al. 2002; Jagiello et al. 2019).

Associated with increasing global human population size, the amount of waste produced is also growing. Globally, three million tonnes of waste are discarded daily (Hoornweg et al. 2013); it is estimated that by 2025, this amount will double to six million tonnes a day (Hoornweg and Bhada-Tata 2012; Hoornweg et al. 2013). In developed countries, 59% of waste is disposed of in landfills (Hoornweg and Bhada-Tata 2012), which, since they contain a constant, abundant, and predictable supply of organic waste, have become a new important source of food for animals globally. To date, 98 different vertebrate species have been observed foraging on landfills worldwide (Plaza and Lambertucci 2017).

In Spain, the implementation of large open landfills at the end of the 1980s has apparently contributed to the recovery of declining by 11% populations of the white stork *Ciconia ciconia* (Senra and Alés 1992)*.* In fact, the reproductive population of white storks in Spain increased by a factor of five between the 1980s and 2004 (Lázaro et al. 1986; Molina and Del Moral 2005), probably because of changes in the species’ migratory behaviour and an increase in the survival rate of juveniles associated with food in open landfills (Gómez-Tejedor and de Lope 1993; Tortosa et al. 1995; Blanco 1996; Tortosa et al. 2002). Similarly, the white stork population in France, which had undergone a marked decline starting in 1960, has increased in western France since 1980 due to reintroduction programmes and the use of landfills as food source (Barbraud et al. 1999).

However, landfills happen to be sources of environmental contimants, such as brominated flame retardants which can negatively affect birds foraging on or near to landfills (Tongue et al. 2019). Moreover, landfills can represent contribution of new sources of anthropogenic nest materials (Witteveen et al. 2017). In avian taxa, the incorporation of durable anthropogenic nest materials (hereafter called ‘debris’) into nests may be considered a consequence of human activities (Wang et al. 2009; Lee et al. 2015). This is consistent with the fact that in an environment with greater human activity, as measured by the HFI, where the probability of debris incorporation into nests is higher (Jagiello et al. 2019), birds incorporate debris intentionally perhaps as a result of scarcity of natural nest materials in human-impacted environments (Antczak et al. 2010; Lee et al. 2015; Reynolds et al. 2019), ectoparasite defence (Suárez-Rodríguez et al. 2012), or signal to conspecifics (Sergio et al. 2011).

Previous studies on white storks indicate that this species incorporates debris, such as plastic string, foil, rubber band into nests (Henry et al. 2011; Jagiello et al. 2018), and forages on landfills (Henry et al. 2011; Plaza and Lambertucci 2017). We suspect that landfills can act as a additional source of replacement of scarce natural materials in highly human impacted environments (as measured by HFI). A previous study showed that white storks collect debris from the vicinity of their nests (Jagiello et al. 2018). Therefore, white stork can be considered a suitable species for investigating the nest content in environments which differ in urbanization level.

In this study, we investigate the incorporation of debris by white storks into their nests along with an urbanization gradient. We aim to test whether human activity (as measured by HFI) is reflected in the frequency (ratio between the number of nests without debris and nests with debris, and then also considered number of pieces of debris and the total mass of debris) of the incorporation of debris into white storks’ nests, and whether this incorporation is related to distance to the nearest landfill. Individuals from environments under greater human activity on environment are predicted to use landfill-derived nest material resources more often, which would be evidenced by greater numbers of nests incorporating human-derived nest materials and in a higher number and total mass of debris in nests. Through this research, we aim to focus more specifically on one species nesting along urbanization gradient, in relation to distance of the nearest landfill.

## Materials and methods

**Fig. 1:**
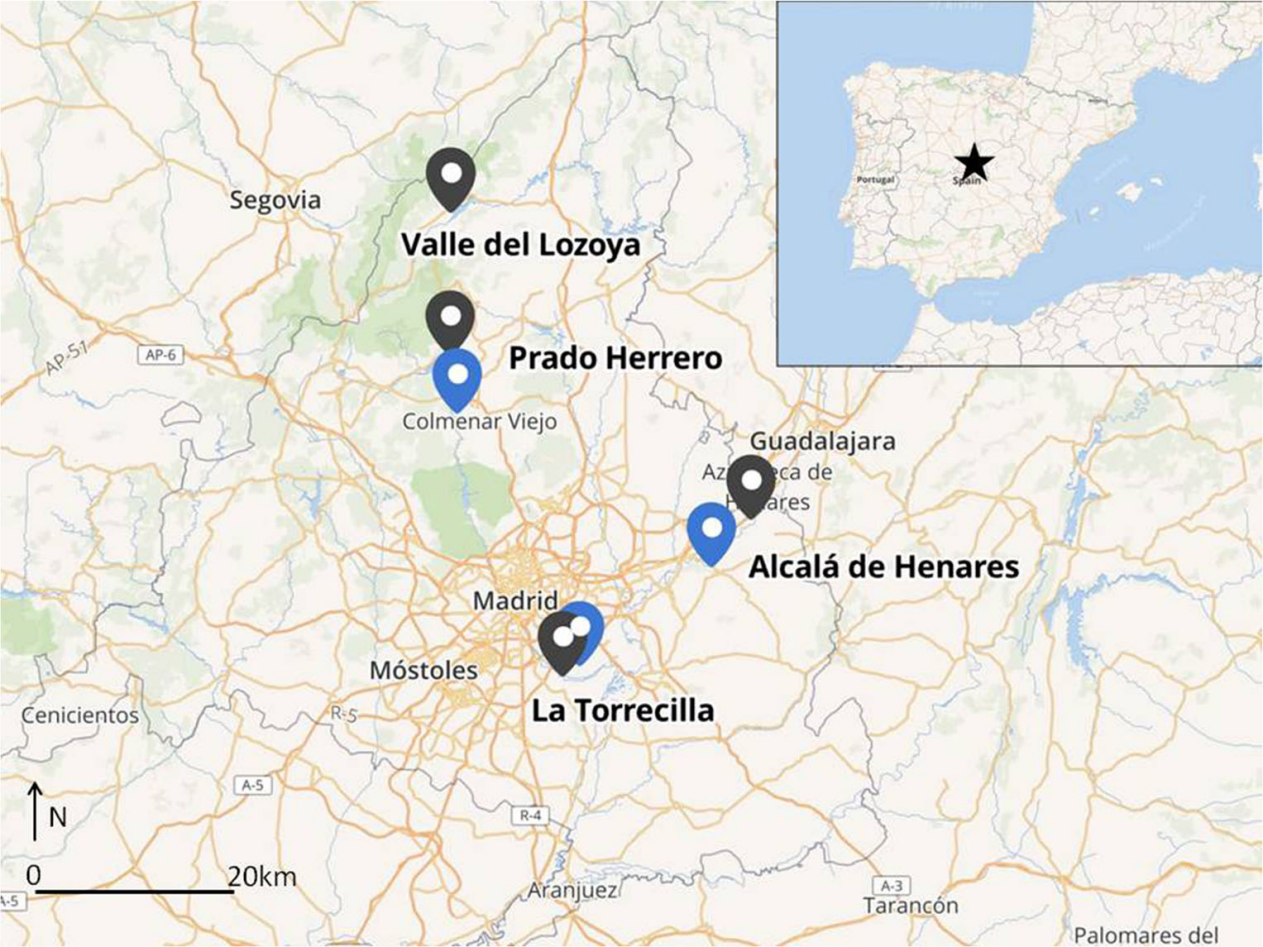
The map showing the locations of the study (black points) and landfills (blue points)

The data were collected in locations around the city of Madrid (Central Spain): (1) La Torrecilla (40°18′N, 3°37′W); (2) Alcalá de Henares (40°29′N, 3°21′W), (3) Prado Herrero (40°44′N, 3°49′W) and (4) Valle del Lozoya (40°55′N, 3°48′W) (Fig. 1). The nests were selected according to the criterion of increasing proximity to the large city of Madrid, ranging from 60 to 0.5 km away. La Torrecilla and Alcalá de Henares are human-altered areas—as measured by HFI (in both locations human activities are high) south of Madrid; the storks nest in the former is on a cattle farm, in the vicinity of Madrid’s biggest landfill, whereas the latter locations is a city with approximately 200,000 inhabitants (Prieto 2002). Prado Herrero is a private cattle farm inside a protected area (Cuenca Alta del Manzanares Regional Park), whereas the Valle del Lozoya is located in an area characterized by ash trees (*Fraxinus angustifolius*), also settled inside a protected area (Guadarrama National Park); both of these localizations are found north-west of Madrid. Prado Herrero and Valle del Lozoya represent low human activities, measured by HFI. Prado Herrero is a part of long-term study on white stork breeding biology. The study sites were selected in order of designing urbanization gradient; what is more, we treated each nest independently, so we calculated the HFI and the distance to the nearest landfill for each nest. Only Prado Herrero and La Torrecilla are white stork colonies, where nests were selected randomly for closer examination. Therefore, for the analysis to control spatial autocorrelation, we added geographical latitude and longitude of nest locations in all models as the interaction of regression splines (Wood 2006).

The data were collected during the 2018 breeding season, within the ringing period for nestlings (between 25 and 45 days of age), exclusively from nests characterized by successful breeding (production of at least one fledgling). We recorded whether there was debris (materials which are from human origin, like plastic, paper, cloth) on the surface of the nest or not, to identify only debris which was brought by storks in the focal breeding season. When present, debris was collected for subsequent analysis. We collected debris when it was possible to remove them from the nest. In some cases (34% from 28 nests), this was not possible because items were firmly attached to the nest structure. For those items, we recorded type (as plastic, cloth, paper, and other) and number of pieces of debris.

We assigned HFI values to each sampled nest. The Global HFI dataset was downloaded from the NASA Socioeconomic Data and Applications Center website (http://sedac.ciesin.columbia.edu/data/set/wildareas-v2-human-footprint-geographic/data-download). The Human Footprint Index was assigned with an accuracy of 1 km^2^. The sampled nest formed the central point of each grid. Whenever sampled nests were found in the same immediate vicinity, normally within a radius of less than 500 m (Jovani and Tella 2007; Molina and Del Moral 2005), the same HFI value was assigned to all of them. In addition, we measured the distance from each nest to the closest landfill, in order to determine whether the vicinity of a landfill affected the number of items and total mass of debris in nests.

### Data analyses

We used general additive models (GAMs) to test relationships between debris incorporation in the nests of white storks and the surrounding environment. For reasons of multicollinearity between explanatory variables: HFI and distance to landfill (VIF > 3), we built separate models for each explanatory variables. We used Z-sore transformation to standardize explanatory variable. To evaluate the effect of HFI (GAM_1) and distance to landfill (GAM_2) on number of pieces of debris in the nest, we used Poisson error structure and log link function (Zuur et al. 2009). To evaluate the effect of HFI (GAM_3) and distance to landfill (GAM_4) on the total mass of debris (natural logarithm transformed), we used Gaussian error structure and identity link function (Zuur et al. 2009). To control for spatial autocorrelation, we added geographical latitude and longitude in all models as the interaction of regression splines (Wood 2006). In all analysis, we used a significance value of *p* < 0.05. The spatial measurements and analyses were performed with QGIS (version 2.18.15) software. The statistical analyses were carried out in R 3.4.3 (R Core Team 2017). The models were calculated in the mgcv package (Wood 2017). Data visualization was carried out in the ggplot2 package (Wickham 2009).

## Results

In total, we examined 49 nests in detail for four locations: La Torrecilla (9), Alcalá de Henares (8), Prado Herrero (25), and Valle del Lozoya (7). In 28 (57% out of 49) nests, we collected debris; in six of these, there were more than 10 items, whereas in 21 nests, there was no debris.

**Fig. 2:**
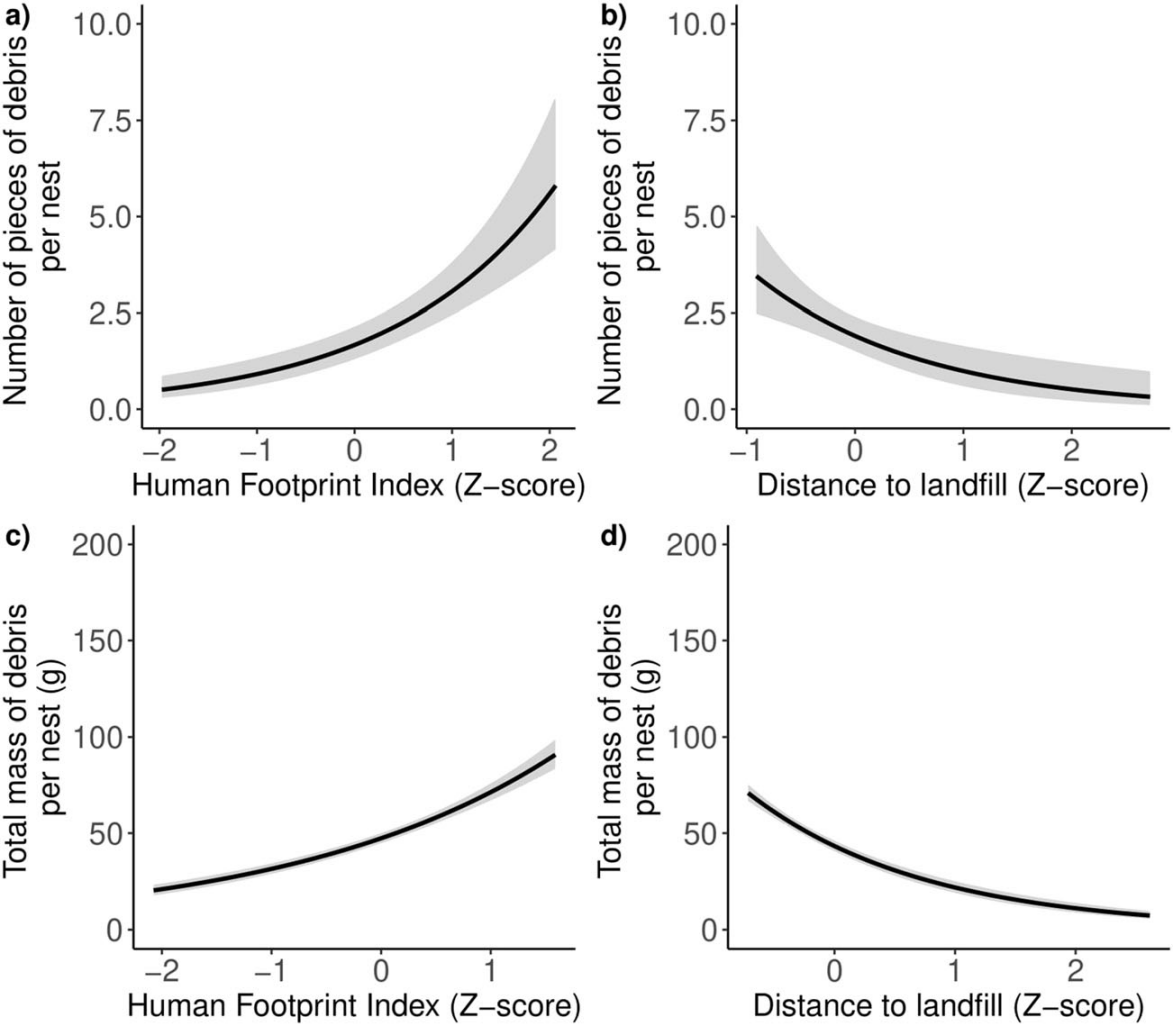
The relationship between number of pieces of debris per nest and (a) the Human Footprint Index (HFI), and (b) distance to landfill, and between the total mass of debris per nest and (c) HFI, and (d) distance to landfill in white storks nesting in Madrid and its environments in 2018. The solid curves are based on cubic splines with SE

The number of pieces of debirs incorporated in the white stork nests was positively related to HFI (Wald χ^2^ = 12.08, *p* = 0.001, Table 1) and negatively related to distance to landfill (Wald χ^2^ = 15.35, *p* = 0.03, Table 1). The graphical visualizations using a cubic spline showed that number of pieces of debirs increased with to HFI (Fig. 2a) in case and decreased with the distance to landfill (Fig. 2b). The total sum of debris weight was significantly negatively related with distance to landfill (F = 2.48, *p* = 0.03, Table 1); however, we did not found significant relationship with HFI (F = 0.49, *p* = 0.10, Table 1, Fig. 2c). The graphical visualizations using a cubic spline showed that total sum of debris weight decrease with the distance to landfill (Fig. 2d).

**Table 1:** Results of the models including the effects of Human Footprint Index and distance to landfill on the number of pieces of debris items and total mass

	edf	Ref.df	Statistic	*p* value
*Number of pieces of debris*				
*GAM 1 R*^*2*^ *= 69.4%*				
Latitude	1.98	2	25.15	< 0.001
Longitude	2.00	2	8.58	0.01
HFI	3.91	9	12.08	0.001
*GAM 2 R*^*2*^ *= 68.7*%				
Latitude	2.00	2	17.40	< 0.001
Longitude	2.00	2	3.01	0.22
Distance to landfill	7.20	9	15.35	0.03
*Total mass of debris*				
*GAM 3 R*^*2*^ *= 61.5%*				
Latitude	1.00	1	20.54	< 0.001
Longitude	1.80	2	1.93	0.16
HFI	1.82	9	0.49	0.10
*GAM 4 R*^*2*^ *= 68.8%*				
Latitude	1.00	1	0.03	0.86
Longitude	1.00	1	0.21	0.66
Distance to landfill	7.97	9	2.48	0.03

*edf* effective degrees of freedom, *Ref.df* reference number of degrees of freedom used for hypothesis testing, Statistic – Chi-square for GAM_1 and GAM_2; *F* for GAM_3 and GAM_4

## Discussion

Our study shows that the incorporation of debris by white storks into their nests is related to human activity (measured by HFI). Moreover, the distance from a stork colony to the nearest landfill appeared to be related to the number of items incorporated into nests.

The number of pieces of debris items in nests increased along with higher values of the Human Footprint Index. High values of the HFI means that environment is highly altered by humans presence and human activities, i.e. human population density, settlements, crop/pasture land, roads, and other access points, night-time lights, and incorporates factors such as the size and remoteness of a given area. As HFI measures human impact, the incorporation of debris into nests represents a response on the part of white storks to human impacted environment. This is consistent with results from a global literature review (Jagiello et al., 2019) and empirical research on this topic (e.g. Wang et al. 2009; Lee et al. 2015). A study on Chinese bulbuls *Pycnonotus sinensis* indicated that the use of debris by members of this species in their nests increased with urbanization (Wang et al. 2009). Interestingly, a study of black-faced spoonbills *Platalea minor* nesting in highly human-altered environment (metropolitan city of Incheon in South Korea) showed that experimental provision of natural nest materials reduced the number of debris items in nests (Lee et al. 2015). We showed, this effect as measured by HFI and relationship to the distance to landfill on populations along a urbaniazation gradient. Moreover, our study concerns terrestrial birds nesting in close proximity; thus data are provided from many nests in the same location, which gives a clearer view of the effect of the environment.

The relationship between the distance from a nesting location to the nearest landfill and the number of debris items in nests was significant. The probable cause of this effect is that individuals nesting in more urbanized environments use landfill-derived nest material resources more often than storks nesting in a less urbanized environment (A. López-García pers. obs.)

The Spanish white stork population has been reported foraging on landfills since the 1980s and 1990s (Gómez-Tejedor and de Lope 1993; Tortosa et al. 1995; Blanco 1996). Moreover, a study of white storks by Henry et al. (2011) revealed that distance from the colony to the landfill was a factor negatively influencing the number of ingested rubber bands, which were being mistaken for prey (earthworms Lumbricidae) and consumed by nestlings.

Changes in the environment such as conversion of land into production land, intensification of agriculuture, and spread of urban areas caused by humans could not only cause food shortage but also limit the availability of natural nest materials (Antczak et al. 2010; Lee et al. 2015). Landfills provide not only new food supplies, which can be harmful (Tongue et al. 2019), but also new nesting materials. We have no proof that storks collect debris directly from landfills, but this is possible, since, when distances from colonies to landfills were smaller, numbers of debris items in nests were higher.

Overall, the incorporation of debris into nests is the white stork’s response to human activity. We observed a positive relationship between white storks nest and in nest’s surrounding area expressed in the number of pieces of debris. Thus, the white stork could serve as a potential indicator of debris pollution in the surrounding environment (Jagiello et al. 2018). Maps of debris (e.g. plastic) pollution/distribution are not available for the terrestrial environment, as opposed to the marine environment (Eriksen et al. 2014). Moreover, the creation of maps concerning terrestrial pollution is strongly advisable (Jagiello et al., 2019), as contamination of plastic in environment is a critical issue on a global scale. Until now, there is only one global project concernig the marine debris and measuring the pollution of the coasts, conducted by CSIRO Marine Debris Research. Highlighting the fact of constant overproduction of waste globally, with growing human populations, the impact of pollution with debris on fauna is expected to grow (Wilcox et al. 2015) in the future. Therefore, composition of white storks’ nests can provide a proxy of nesting behaviour of other bird species in close relation to humans in reponse to anthropogenic activities.

## References

[CR1] Antczak M, Hromada M, Czechowski P, Tabor J, Zabłocki P, Grzybek J, Tryjanowski P (2010). A new material for old solutions – the case of plastic string used in great grey shrike nests. Acta Ethol.

[CR2] Barbraud C, Barbraud J, Barbraud M (1999). Population dynamics of the white stork *Ciconia ciconia* in western France. Ibis.

[CR3] Blanco G (1996). Population dynamics and communal roosting of white storks foraging at a Spanish refuse dump. Colon Waterbirds.

[CR4] Cardinale BJ, Duffy JE, Gonzalez A, Hooper DU, Perrings C, Venail P, Narwani A, Mace GM, Tilman D, Wardle DA, Kinzig AP, Daily GC, Loreau M, Grace JB, Larigauderie A, Srivastava DS, Naeem S (2012). Biodiversity loss and its impact on humanity. Nature.

[CR5] Eriksen M, Lebreton LCM, Carson HS, Thiel M, Moore CJ, Borerro JC, Galgani F, Ryan PG, Reisser J (2014). Plastic pollution in the world’s oceans: more than 5 trillion plastic pieces weighing over 250,000 tons afloat at sea. PLoS One.

[CR6] Gómez-Tejedor H, de Lope F (1993). Sucesión fenológica de las aves no passeriformes en el vertedero de Badajoz. Ecología.

[CR7] Henry PY, Wey G, Balança G (2011). Rubber band ingestion by a rubbish dump dweller, the white stork (*Ciconia ciconia*). Waterbirds.

[CR8] Hooper DU, Adair EC, Cardinale BJ, Byrnes JEK, Hungate BA, Matulich KL, Gonzalez A, Duffy JE, Gamfeldt L, O'Connor MI (2012). A global synthesis reveals biodiversity loss as a major driver of ecosystem change. Nature.

[CR9] Hoornweg D., Bhada-Tata P. (2012) What a Waste : A Global Review of Solid Waste Management. Urban development series;knowledge papers no. 15. World Bank, Washington, DC. © World Bank. https://openknowledge.worldbank.org/handle/10986/17388 License: CC BY 3.0 IGO

[CR10] Hoornweg D, Bhada-Tata P, Kennedy C (2013). Waste production must peak this century. Nature.

[CR11] Jagiello ZA, Dylewski Ł, Winiarska D, Żołnierowicz KM, Tobółka M (2018). Factors determining the occurrence of anthropogenic materials in nests of the white stork *Ciconia ciconia*. Environ Sci Pollut Res.

[CR12] Jagiello Z, Dylewski Ł, Tobolka M, Aguirre JI (2019) Life in a polluted world: A global review of anthropogenic materials in bird nests. Environ Pollut 251:717–722. 10.1016/j.envpol.2019.05.028 10.1016/j.envpol.2019.05.028 31108305

[CR13] Jones KR, Venter O, Fuller RA, Allan JR, Maxwell SL, Negret PJ, Watson JEM (2018). One-third of global protected land is under intense human pressure. Science.

[CR14] Jovani R, Tella JL (2007). Fractal bird nest distribution produces scale-free colony sizes. Proc Biol Sci.

[CR15] Kalnay E, Cai M (2003). Impact of urbanization and land-use change on climate. Nature.

[CR16] Lázaro E, Chozas P, Fernandéz-Cruz M (1986). Demografía de la cigüeña blanca (*Ciconia ciconia*) en España. Censo Nacional de 1984. Ardeola.

[CR17] Lee K, Jang YC, Hong S, Lee J, Kwon IK (2015). Plastic marine debris used as nesting materials of the endangered species black-faced spoonbill *Platalea minor* decreases by conservation activities. J Korean Soc Mar Environ Energy.

[CR18] Lowe S, Browne M, Boudjelas S, De Poorter M (2000) 100 of the World’s Worst Invasive Alien Species A selection from the Global Invasive Species Database. Published by The Invasive Species Specialist Group (ISSG) a specialist group of the Species Survival Commission (SSC) of the World Conservation Union (IUCN), 12pp. First published as special lift-out in Aliens 12 December 2000.Updated and reprinted version: November 2004

[CR19] Lowry H, Lill A, Wong BMB (2012). Behavioural responses of wildlife to urban environments. Biol Rev.

[CR20] Molina B, Del Moral JC (2005) La cigüeña blanca en España. VI Censo de Internacional (2004) (SEO/Birdlife, ed.). Madrid: SEO/Birdlife

[CR21] Plaza PI, Lambertucci SA (2017). How are garbage dumps impacting vertebrate demography, heath, and conservation?. Glob Ecol Conserv.

[CR22] Prieto J (2002) Las Cigüeñas de Alcalá. Alcalá de Henares: Escuela Taller de Medio Ambiente Albardín. Parque de los Cerros

[CR23] R Core Team Development (2017) R: a language and environment for statistical computing. R Foundation for Statistical Computing, Vienna

[CR24] Reynolds SJ, Ibáñez-Álamo JD, Sumasgutner P, Mainwaring MC (2019). Urbanisation and nest building in birds: a review of threats and opportunities. J Ornithol.

[CR25] Sanderson EW, Jaiteh M, Levy MA, Redford KH, Wannebo AV, Woolmer G (2002). The human footprint and the last of the wild: the human footprint is a global map of human influence on the land surface, which suggests that human beings are stewards of nature, whether we like it or not. BioScience.

[CR26] Senra A, Alés EE (1992). The decline of the white stork Ciconia ciconia population of western Andalusia between 1976 and 1988: causes and proposals for conservation. Biol Conserv.

[CR27] Sergio F, Blas J, Blanco G, Tanferna A, Lopez L, Lemus JA, Hiraldo F (2011). Raptor nest decorations are a reliable threat against conspecifics. Science.

[CR28] Suárez-Rodríguez M, Lopez-Rull I, Macias Garcia C (2012). Incorporation of cigarette butts into nests reduces nest ectoparasite load in urban birds: new ingredients for an old recipe?. Biol Lett.

[CR29] Tongue A, Reynolds SJ, Fernie KJ, Harrard S (2019). Flame retardant concentrations and profiles in wild birds associated with landfill: a critical review. Environ Pollut.

[CR30] Tortosa FS, Manez M, Barcell M (1995). Wintering white storks (*Ciconia ciconia*) in south west Spain in the years 1991 and 1992. Die Vogelwarte.

[CR31] Tortosa FS, Caballero JM, Reyes-Lopez J (2002). Effect of rubbish dumps on breeding success in the white stork in southern Spain. Waterbirds.

[CR32] Venter O, Sanderson EW, Magrach A, Allan JR, Beher J, Jones KR, Possingham HP, Laurance WF, Wood P, Fekete BM, Levy MA, Watson JEM (2016). Sixteen years of change in the global terrestrial human footprint and implications for biodiversity conservation. Nat Commun.

[CR33] Walther GR, Post E, Convey P, Menzel A, Parmesan C, Beebee TJ, Fromentin JM, Hoegh-Guldberg O, Bairlein F (2002). Ecological responses to recent climate change. Nature.

[CR34] Wang Y, Chen S, Blair RB, Jiang P, Ding P (2009). Nest composition adjustments by Chinese bulbuls (*Pycnonotus sinensis*) in an urbanized landscape of Hangzhou (E China). Acta Ornithol.

[CR35] Wickham H (2009). ggplot2: Elegant Graphics for Data Analysis.

[CR36] Wilcox C, Van Sebille E, Hardesty BD (2015). Threat of plastic pollution to seabirds is global, pervasive, and increasing. Proc Natl Acad Sci U S A.

[CR37] Witteveen M, Brown M, Ryan PG (2017). Anthropogenic debris in the nests of kelp gulls in South Africa. Mar Pollut Bull.

[CR38] Wood SN (2006). Low-rank scale-invariant tensor product smooths for generalized additive mixed models. Biometrics.

[CR39] Wood S (2017) Generalized Additive Models: An Introduction with R, 2 edition. Chapman and Hall/CRC

[CR40] Zuur A, Ieno EN, Walker N, Saveliev AA, Smith GM (2009) Mixed effects models and extensions in ecology with R. Springer Science & Business Media

